# Impact of DNA Sequencing and Analysis Methods on 16S rRNA Gene Bacterial Community Analysis of Dairy Products

**DOI:** 10.1128/mSphere.00410-18

**Published:** 2018-10-17

**Authors:** Zhengyao Xue, Mary E. Kable, Maria L. Marco

**Affiliations:** a Department of Food Science & Technology, University of California, Davis, California, USA; University of Wisconsin—Madison

**Keywords:** 16S rRNA, DNA sequencing, dairy, microbiome, microbiota, milk

## Abstract

DNA sequencing and analysis methods were compared for 16S rRNA V4 PCR amplicon and genomic DNA (gDNA) mock communities encompassing nine bacterial species commonly found in milk and dairy products. The two communities comprised strain-specific DNA that was pooled before (gDNA) or after (PCR amplicon) the PCR step. The communities were sequenced on the Illumina MiSeq and Ion Torrent PGM platforms and then analyzed using the QIIME 1 (UCLUST) and Divisive Amplicon Denoising Algorithm 2 (DADA2) analysis pipelines with taxonomic comparisons to the Greengenes and Ribosomal Database Project (RDP) databases. Examination of the PCR amplicon mock community with these methods resulted in operational taxonomic units (OTUs) and amplicon sequence variants (ASVs) that ranged from 13 to 118 and were dependent on the DNA sequencing method and read assembly steps. The additional 4 to 109 OTUs/ASVs (from 9 OTUs/ASVs) included assignments to spurious taxa and sequence variants of the 9 species included in the mock community. Comparisons between the gDNA and PCR amplicon mock communities showed that combining gDNAs from the different strains prior to PCR resulted in up to 8.9-fold greater numbers of spurious OTUs/ASVs. However, the DNA sequencing method and paired-end read assembly steps conferred the largest effects on predictions of bacterial diversity, with effect sizes of 0.88 (Bray-Curtis) and 0.32 (weighted Unifrac), independent of the mock community type. Overall, DNA sequencing performed with the Ion Torrent PGM and analyzed with DADA2 and the Greengenes database resulted in the most accurate predictions of the mock community phylogeny, taxonomy, and diversity.

**IMPORTANCE** Validated methods are urgently needed to improve DNA sequence-based assessments of complex bacterial communities. In this study, we used 16S rRNA PCR amplicon and gDNA mock community standards, consisting of nine, dairy-associated bacterial species, to evaluate the most commonly applied 16S rRNA marker gene DNA sequencing and analysis platforms used in evaluating dairy and other bacterial habitats. Our results show that bacterial metataxonomic assessments are largely dependent on the DNA sequencing platform and read curation method used. DADA2 improved sequence annotation compared with QIIME 1, and when combined with the Ion Torrent PGM DNA sequencing platform and the Greengenes database for taxonomic assignment, the most accurate representation of the dairy mock community standards was reached. This approach will be useful for validating sample collection and DNA extraction methods and ultimately investigating bacterial population dynamics in milk- and dairy-associated environments.

## Abstract (precis)

Validated methods are urgently needed to improve DNA sequence-based assessments of complex bacterial communities. In this study, we used 16S rRNA PCR amplicon and gDNA mock community standards, consisting of nine, dairy-associated bacterial species, to evaluate the most commonly applied 16S rRNA marker gene DNA sequencing and analysis platforms used in evaluating dairy and other bacterial habitats. Our results show that bacterial metataxonomic assessments are largely dependent on the DNA sequencing platform and read curation method used. DADA2 improved sequence annotation compared with QIIME 1, and when combined with the Ion Torrent PGM DNA sequencing platform and the Greengenes database for taxonomic assignment, the most accurate representation of the dairy mock community standards was reached. This approach will be useful for validating sample collection and DNA extraction methods and ultimately investigating bacterial population dynamics in milk- and dairy-associated environments.

## INTRODUCTION

Advancements in massively parallel DNA sequencing technologies have resulted in a dramatic increase in knowledge of the microorganisms found in natural environments, food systems, and the human body. 16S rRNA gene amplicon sequencing, in particular, has been a cornerstone for investigating bacterial diversity and phylogeny. This approach has enabled the simultaneous identification of the majority of bacteria in complex microbial communities. Although analysis of 16S rRNA gene diversity has provided significant new perspectives on bacterial habitats, there remain challenges to sample preparation, DNA sequencing, and data analysis approaches for ensuring accurate measurements of bacterial populations.

To address these issues, recent studies have compared sample collection methods (1–3) and storage conditions (2, 4–9). These studies generally showed that differences in bacterial composition caused by those methodological alterations are relatively minor compared to intersample variation (1, 3, 5–9). DNA extraction methods, on the other hand, can result in major changes to estimates of bacterial proportions between Gram-positive and Gram-negative bacteria, which are more or less difficult to lyse (4, 7, 10–15). Moreover, PCR can also introduce bias depending on the DNA polymerase (16), number of cycles (17), and variable region of the 16S rRNA gene being compared (4, 18, 19).

DNA sequencing platforms, including 454 pyrosequencing, Illumina, Ion Torrent, and Pacific Biosciences have also been shown to cause variation in bacterial community assessments (4, 18–21). Moreover, data analysis methods, especially read clustering approaches (i.e., generating representative sequences), are known to have a significant impact on the interpretation of bacterial composition (21–27). *De novo* sequence clustering can result in unstable operational taxonomic units (OTUs) between projects that are composed of different sequences with each clustering iteration (28, 29). Reference-based sequence clustering tends to result in fewer sequence variants than *de novo* methods (21, 23), but can still lead to overestimation of bacterial community diversity caused by insufficient read quality control and error filtering (27). As a result, there is now an effort to move away from OTU-based methods toward DNA sequences that represent single nucleotide variation (30, 31). One of the amplicon sequence variant (ASV) clustering methods is DADA2 (Divisive Amplicon Denoising Algorithm 2), which builds a quality-based model for filtering error and identifying variation in 16S rRNA gene sequences (26).

**FIG 1:**
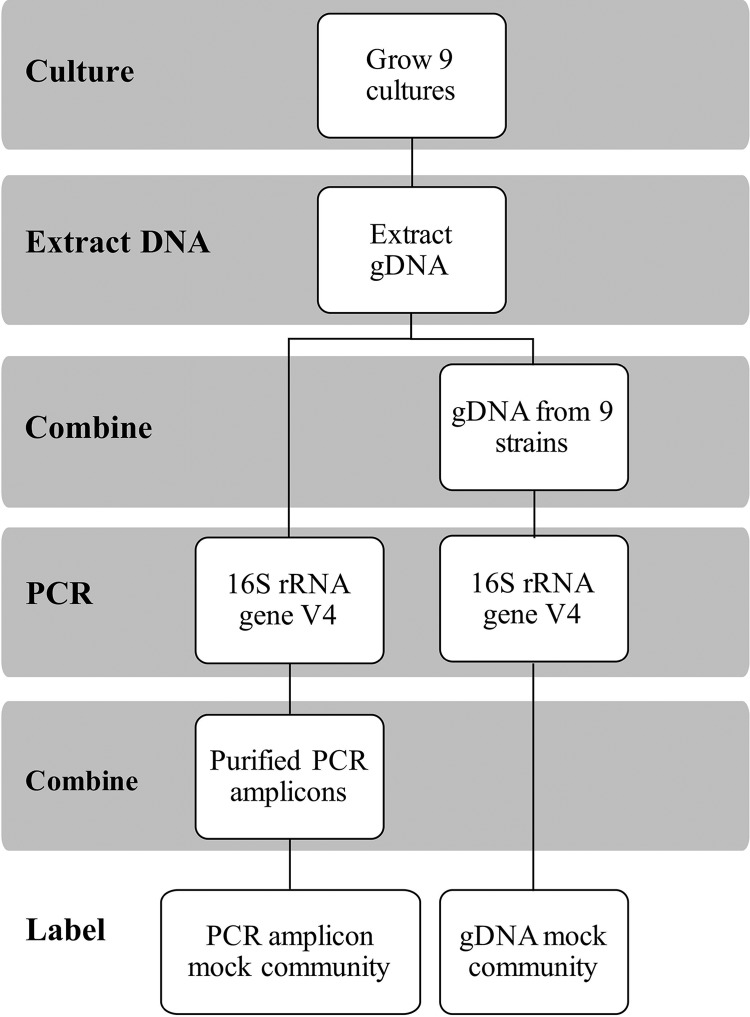
Schematic diagram of the experimental design. Genomic DNAs were individually prepared from nine bacterial broth cultures, purified, and combined for the gDNA mock community. Additionally, each gDNA was amplified separately and pooled for the PCR amplicon mock community.

Herein, we sought to compare different DNA sequencing, read assembly, and data analysis strategies for the capacity to accurately detect the composition of a mock bacterial community consisting of nine species commonly found in milk. To eliminate biases introduced by sample type and DNA extraction method and focus on close examination of the biases introduced by PCR, sequencing, and bioinformatics analyses, we employed two different mock communities consisting of either organism-specific PCR amplicons or purified genomic DNA (gDNA) (Fig. 1). This approach allowed us to compare the performance of two popular benchtop DNA sequencers (Illumina MiSeq and Ion Torrent PGM), paired-end read assembly of Illumina MiSeq, OTU (QIIME 1 open reference)/ASV (DADA2) analysis methods, and reference taxonomy databases (Greengenes and RDP). Our results showed that the combination of DADA2 and the Greengenes analysis pipeline, paired with Ion Torrent PGM sequencing, results in the most accurate representation of the mock communities.

## RESULTS

### Comparison of representative sequence analysis of 16S rRNA V4 region reads generated with the Illumina MiSeq and Ion Torrent PGM.

A mock community was prepared by combining 16S rRNA V4 region PCR amplicons from nine bacterial strains (Table 1) in equimolar quantities prior to DNA sequencing on the Ion Torrent PGM and Illumina MiSeq instruments. Sequences were either assembled (Illumina MiSeq) or maintained as single-end reads (Illumina MiSeq and Ion Torrent PGM). A low percentage of reads were identified as chimeras (0 to 1.4%) (see Table S1 in the supplemental material), and the remaining reads were analyzed using QIIME 1 (UCLUST) following the open-reference pipeline at a 97% threshold or DADA2 pipelines for OTU or ASV identification using the Greengenes (version 13.8) and RDP (version GOLD for QIIME 1 and version 11.5 for DADA2) reference databases. Total reads after quality filtering for each sequencing and read assembly method are shown in Table S1.

**TABLE 1:** Bacterial strains and expected relative abundances in the gDNA mock community

Strain	No. of 16S rRNA gene copies^*a*^	% of total^*b*^	Genome reference^*c*^
Bacillus subtilis S44	10	16.67	NA
Clostridium tyrobutyricum ATCC 25755	6	2.29	64
Corynebacterium bovis ATCC 7715	1	2.78	65
Enterococcus faecalis ATCC 29212	4	9.28	66
Escherichia coli ATCC 700728	7	8.95	NA
Lactococcus lactis IL1403	6	17.82	67
Pseudomonas fluorescens A506	6	7.08	68
Staphylococcus aureus ATCC 29740	5	12.44	NA
Streptococcus agalactiae ATCC 27956	7	22.7	NA

a Number of 16S rRNA gene copies per genome based on genome reference.

b Percentage of total bacterial 16S rRNA gene in the mock community according to DNA concentration.

c NA, not available. For strains that lack whole-genome sequences, the genome sizes and 16S rRNA gene copy numbers of the reference strain were used (69–72).

10.1128/mSphere.00410-18.8 TABLE S1 Quality-filtered read numbers and chimeras found for the mock communities. Download Table S1, PDF file, 0.1 MB. Copyright © 2018 Xue et al. 2018 Xue et al. This content is distributed under the terms of the Creative Commons Attribution 4.0 International license.

### Illumina MiSeq paired-end assemblies.

QIIME 1 analysis of Illumina MiSeq paired-end assembled reads with recommended parameters (32) resulted in total OTU numbers that were at least 4.2-fold greater than the expected nine OTUs encompassing strains included in the mock community (Table 2). When the Greengenes database was used for OTU alignment, 85 OTUs were identified. The majority of those OTUs (i.e., 65) were assigned to taxa included in the mock community. Although the numbers of OTUs varied for each taxon, a single OTU representative encompassed the majority (> 60%) of reads for each of the mock community members (Table 2). For example, out of nine Staphylococcus OTUs identified with Greengenes, 99% of the reads were represented by one OTU. The remaining 20 OTUs identified with Greengenes were either designated as taxa that were not included in the mock community or were designated only to the order level. When the RDP database was used as the reference database for QIIME 1 analysis, the total OTUs decreased to 70, despite the increase in spurious “other” assignments (Table 2). Nevertheless, the overall OTU number remained considerably higher than the expected nine OTUs based on the mock community composition.

**TABLE 2:** OTU/ASV distribution of the 16S rRNA PCR amplicon mock community following Illumina MiSeq DNA sequencing and paired-end assembly

Taxonomy	No. (%) of OTUs/ASVs by^*a*^:			
	QIIME 1		DADA2	
	Greengenes	RDP	Greengenes	RDP
Bacillaceae		1 (100)		
Bacillus	10 (83)	3 (99)	2 (90)	2 (90)
Clostridiaceae	3 (73)	3 (51)	1 (100)	
Clostridium	3 (87)	5 (99)	4 (86)	5 (85)
Corynebacterium	14 (80)	7 (80)	2 (91)	2 (91)
Enterococcaceae		1 (100)		
Enterococcus	4 (99)	2 (99)	3 (85)	3 (85)
Enterobacteriaceae	4 (99)	9 (71)		2 (88)
Escherichia			2 (88)	
Lactococcus	6 (99)	1 (100)	3 (89)	3 (89)
Pseudomonas	10 (74)	6 (75)	3 (85)	3 (85)
Staphylococcus	9 (99)	4 (80)	3 (89)	3 (89)
Streptococcus	2 (80)	2 (99)	2 (89)	2 (89)
Other	20 (17)	26 (71)	13 (20)	13 (20)
Sum	85	70	38	38

a Each value represents the average number of OTUs/ASVs (*n* = 3) and mean percentage of sequence reads assigned to the most abundant OTU/ASV within that taxon.

The numbers of ASVs identified with DADA2 from Illumina paired-end assemblies were lower than the numbers of OTUs assigned with QIIME 1 (Table 2). Because DADA2 assigns ASVs independently from taxonomic reference databases, total ASV numbers were the same using both Greengenes and RDP. The only distinction was that one Clostridiaceae ASV and both Escherichia ASVs identified using Greengenes were designated as Clostridium and Enterobacteriaceae in RDP (Table 2).

### Illumina MiSeq unassembled single-end reads.

Without read assembly, the QIIME 1 pipeline resulted in 68 and 36 total OTUs with the Greengenes and RDP databases, respectively (Table 3). These OTU numbers were lower than the paired-end assemblies (Table 2). DADA2, on the other hand, resulted in a slightly higher number of ASVs (40 ASVs) than the paired-end assemblies (38 ASVs) (Tables 2 and 3). More reads were regarded as “other” taxa, and this result was most likely due to the shorter lengths of the unassembled, single-end MiSeq reads. Between the two reference databases, Greengenes resulted in more accurate taxonomic assignments with DADA2. Two Bacillales ASVs and one Clostridiales ASV that were included in the “other” ASV category by RDP were assigned as Bacillus and Clostridiaceae with Greengenes. Moreover, the Escherichia/Shigella ASV in RDP was unambiguously allotted to the Escherichia genus by Greengenes (Table 3).

**TABLE 3:** OTU/ASV distribution of the 16S rRNA PCR amplicon mock community following Illumina MiSeq DNA sequencing without paired-end assembly

Taxonomy	No. (%) of OTUs/ASVs by^*a*^:			
	QIIME 1		DADA2	
	Greengenes	RDP	Greengenes	RDP
Bacillus	9 (97)	2 (99)	3 (67)	1 (100)
Clostridiaceae	1 (100)	1 (100)	1 (100)	
Clostridium	3 (99)	3 (99)	3 (99)	3 (99)
Corynebacterium	9 (96)	7 (95)	2 (75)	2 (75)
Enterococcaceae	1 (100)			
Enterococcus	2 (99)	2 (99)	2 (60)	2 (60)
Enterobacteriaceae	4 (99)	8 (93)		
Escherichia/Shigella				1 (100)
Escherichia			1 (100)	
Lactococcus	6 (99)	2 (99)	1 (100)	1 (100)
Pseudomonas	11 (91)	4 (99)	2 (65)	2 (65)
Staphylococcus	12 (98)	3 (99)	2 (76)	2 (76)
Streptococcus	4 (92)	2 (99)	1 (100)	1 (100)
Other	6 (28)	2 (91)	22 (12)	25 (89)
Sum	68	36	40	40

a Each value represents the average number of OTUs/ASVs (*n* = 3) and mean percentage of sequence reads assigned to the most abundant OTU/ASV within that taxon.

### Ion Torrent PGM reads.

The application of QIIME 1 with the Greengenes database to the Ion Torrent reads resulted in the highest number of OTUs out of any of the methods applied (Table 4). The use of RDP in QIIME 1 also yielded high OTU numbers, comparable to those found for the paired-end Illumina MiSeq assemblies (Table 4). Conversely, DADA2 resulted in only 13 ASVs (Table 4). The 4 additional ASVs compared to the expected 9 ASVs were created due to errors in the homopolymer regions (see Fig. S1 in the supplemental material), and the 13 ASVs were distributed across the 9 bacterial taxa included in the mock community, with the exception of one ASV with ambiguous taxonomy (Bacillales) identified using RDP, which was identified as Bacillus using Greengenes. Greengenes also improved the assignment of Escherichia coli to the genus level, as opposed to the family level in RDP (Table 4).

**TABLE 4:** OTU/ASV distribution of the 16S rRNA PCR amplicon mock community following Ion Torrent PGM sequencing

Taxonomy	No. (%) of OTUs/ASVs by^*a*^:			
	QIIME 1		DADA2	
	Greengenes	RDP	Greengenes	RDP
Bacillaceae	3 (88)			
Bacillus	21 (75)	8 (95)	2 (95)	1 (100)
Clostridium	5 (70)	7 (99)	1 (100)	1 (100)
Corynebacterium	15 (75)	11 (88)	1 (100)	1 (100)
Enterococcaceae	2 (56)	2 (50)		
Enterococcus	7 (98)	2 (99)	1 (100)	1 (100)
Enterobacteriaceae	13 (95)	17 (60)		1 (100)
Escherichia			1 (100)	
Lactococcus	6 (99)	1 (100)	2 (99)	2 (99)
Pseudomonas	13 (71)	8 (66)	2 (99)	2 (99)
Staphylococcus	21 (97)	7 (65)	2 (99)	2 (99)
Streptococcus	6 (83)	2 (99)	1 (100)	1 (100)
Other	6 (40)	2 (71)	0	1 (100)
Sum	118	67	13	13

a Each value represents the average number of OTUs/ASVs (*n* = 3) and mean percentage of sequence reads assigned to the most abundant OTU/ASV within that taxon.

10.1128/mSphere.00410-18.1 FIG S1 DNA sequence alignment of the four taxa with additional ASV sequences identified from Ion Torrent PGM results. ASV sequences were assigned by the DADA2/Greengenes analysis pipeline. Number 1 represents the most abundant ASV out of three replicates for each bacterial taxa included in the mock community. Number 2 represents the low-abundance additional ASVs for each bacterial taxon. The ASV sequences and abundance profiles were the same between the PCR amplicon and gDNA mock communities. Download FIG S1, TIF file, 2.7 MB. Copyright © 2018 Xue et al. 2018 Xue et al. This content is distributed under the terms of the Creative Commons Attribution 4.0 International license.

For each of the three DNA sequencing/read curation methods tested, DADA2 assigned fewer ASVs per taxon and resulted in fewer spurious ASVs than QIIME 1 (UCLUST) assigned OTUs, except in Illumina single-end results analyzed with the RDP database. DADA2 taxonomic identification was more specific with the Greengenes than the RDP database. Therefore, the combined DADA2/Greengenes approach was used for the subsequent analyses described below.

### Assessments of the gDNA mock community were altered depending on DNA sequencing platform.

A gDNA mock community was prepared by mixing equal quantities of gDNA from the nine milk-associated bacterial species prior to barcoded 16S rRNA V4 region PCR amplification (Fig. 1). The PCR products were then used for sequencing on either the Illumina MiSeq or Ion Torrent PGM, followed by analysis with the DADA2/Greengenes method. More chimeras were found for the gDNA mock community (ranging from 0.3 to 4.6%) than the PCR amplicon mock community (Table S1), indicating amplification errors arose from multitemplate PCR. However, except for the known variation in platform-dependent read lengths, nucleotide sequences of the most abundant ASVs assigned to each of the nine mock community species were identical between the Illumina MiSeq (single and paired ends) and Ion Torrent PGM platforms (see Illumina MiSeq paired-end assembly in Fig. S2, Illumina MiSeq single-end reads in Fig. S3, and Ion Torrent PGM reads in Fig. S4 in the supplemental material). Nucleotide sequence alignments of those ASVs to the corresponding ASVs identified from the PCR amplicon mock community also showed 100% nucleotide sequence conservation (Fig. S2 to S4).

10.1128/mSphere.00410-18.2 FIG S2 DNA sequence alignment of the most abundant ASV sequences identified from the Illumina MiSeq paired-end assembly. ASV sequences were assigned by the DADA2/Greengenes analysis pipeline. The most abundant ASV out of three replicates is shown for each bacterial taxon included in the mock community. Download FIG S2, TIF file, 2.2 MB. Copyright © 2018 Xue et al. 2018 Xue et al. This content is distributed under the terms of the Creative Commons Attribution 4.0 International license.

10.1128/mSphere.00410-18.3 FIG S3 DNA sequence alignment of the most abundant ASV sequences identified from the Illumina MiSeq single-end, unassembled reads. ASV sequences were assigned by the DADA2/Greengenes analysis pipeline. The most abundant ASV out of three replicates is shown for each bacterial taxon included in the mock community. DNA alignment length differences were due to read length differences of the forward (196 bases) and reverse (121 bases) reads. Download FIG S3, TIF file, 1.6 MB. Copyright © 2018 Xue et al. 2018 Xue et al. This content is distributed under the terms of the Creative Commons Attribution 4.0 International license.

10.1128/mSphere.00410-18.4 FIG S4 DNA sequence alignment of the most abundant ASV sequences identified from the Ion Torrent PGM results. ASV sequences were assigned by the DADA2/Greengenes analysis pipeline. The most abundant ASV out of three replicates is shown for each bacterial taxon included in the mock community. Download FIG S4, TIF file, 1.9 MB. Copyright © 2018 Xue et al. 2018 Xue et al. This content is distributed under the terms of the Creative Commons Attribution 4.0 International license.

**FIG 2:**
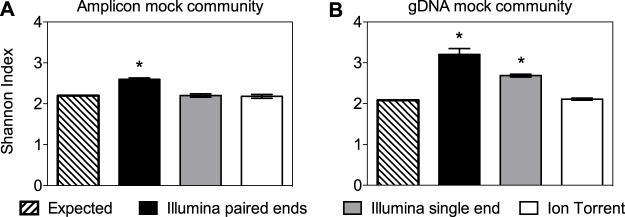
α diversity measurements of mock community samples. Shown is the Shannon index of (A) the PCR amplicon mock community and (B) the gDNA mock community. The results shown were analyzed following the DADA2 pipeline and Greengenes database. Each bar represents the mean ± standard deviation (SD) from three replicates. α diversity measurements for each community were compared to expected values using ANOVA with Bonferroni’s multiple-comparison test. *P* values of <0.05 were considered to be significantly different from the expected values and are indicated by an asterisk above each bar plot.

For both Illumina MiSeq assembled and unassembled (single-end) reads, the gDNA mock community resulted in high numbers of ASVs (Table 5). These numbers were higher than those found for the PCR amplicon mock community (Tables 2 and 3) and were primarily due to the higher quantities of spurious ASVs (e.g., Clostridiales, Lactobacillus, and Oscillospira) present at low proportions (0.02 to 3.85% of total reads for each ASV) (see Table S2 in the supplemental material). As a result, the Shannon index of the gDNA mock community was elevated compared to the PCR amplicon mock community for both paired-end and single-end Illumina MiSeq results (Fig. 2), and these values were significantly increased compared to the expected α diversity based on mock community composition. Interestingly, the same number of 13 ASVs was found for the gDNA and PCR amplicon mock communities when the Ion Torrent PGM was used (Table 5), and the Shannon index of the gDNA mock community resembled the PCR amplicon mock community expected value (Fig. 2).

**TABLE 5:** ASV distribution of the gDNA mock community following different sequencing methods^*a*^

Taxonomy	No. (%) of OTUs/ASVs by^*b*^:		
	Illumina		Ion Torrent
	Paired end	Single end	
Bacillus	4 (86)	2 (96)	2 (95)
Clostridiaceae	3 (92)	1 (100)	
Clostridium	4 (80)	2 (94)	1 (100)
Corynebacterium	2 (96)	1 (100)	1 (100)
Enterococcus	3 (88)	1 (100)	1 (100)
Escherichia	2 (96)	1 (100)	1 (100)
Lactococcus	3 (88)	3 (67)	2 (99)
Pseudomonas	3 (94)	1 (100)	2 (99)
Staphylococcus	2 (90)	2 (99)	2 (99)
Streptococcus	3 (90)	2 (99)	1 (100)
Other	116 (12)	111 (10)	0
Sum	145	127	13

a Results are based on the DADA2 analysis pipeline with the Greengenes database.

b Each value represents the average number of OTUs/ASVs (*n* = 3) and mean percentage of sequence reads assigned to the most abundant OTU/ASV within that taxon.

10.1128/mSphere.00410-18.9 TABLE S2 Percentage and BLASTn results of spurious and ambiguous ASVs and in the gDNA mock community following Illumina Miseq DNA sequencing. Download Table S2, PDF file, 0.1 MB. Copyright © 2018 Xue et al. 2018 Xue et al. This content is distributed under the terms of the Creative Commons Attribution 4.0 International license.

### Ion Torrent PGM sequencing with the DADA2/Greengenes method resulted in more accurate representations of the gDNA and PCR amplicon mock communities.

**FIG 3:**
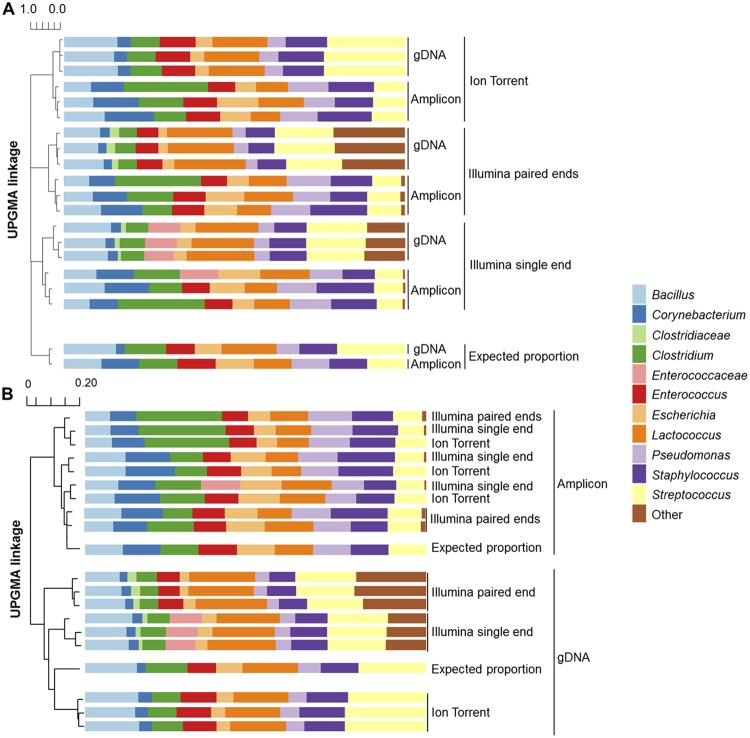
Relative proportions of taxa and UPGMA hierarchical clustering of the mock communities. UPGMA hierarchical clustering was based on the (A) Bray-Curtis dissimilarity matrix and (B) weighted Unifrac distance matrix. Expected taxa (9 bacterial species) are labeled with the corresponding taxonomic level from the DNA sequencing results. Each bar contains the results from each of the three mock community replicates tested using different DNA sequencing methods. The results shown were analyzed following the DADA2 pipeline with the Greengenes database.

DNA sequencing approaches were next compared for their capacity to yield the expected β diversity and proportions of bacterial taxa included in the two mock communities. According to UPGMA (unweighted pair group method using average linkages) hierarchical clustering of Bray-Curtis dissimilarity metrics, results from the three DNA sequencing approaches (Illumina MiSeq paired-end assembly, single-end, and Ion Torrent PGM) were all in different clusters compared to the expected bacterial composition, independent of the gDNA or 16S rRNA PCR amplicon community type (Fig. 3A). Conversely, UPGMA of the weighted Unifrac distance metrics clustered the sequences according to mock community type (Fig. 3B). These comparisons showed that the gDNA mock communities sequenced with the Ion Torrent PGM were the most similar to theoretical (expected) proportions. No single method was found best suited for representing the PCR amplicon mock community (Fig. 3B). To assess whether the use of DADA2/Greengenes influenced this outcome, the other data analysis methods were compared, and it was found that the DNA sequencing platform used was consistently influential on mock community β diversity (e.g., QIIME 1 with the Greengenes database is shown in Fig. S5 in the supplemental material).

10.1128/mSphere.00410-18.5 FIG S5 Principal-coordinate analysis of mock community samples. Shown are results from principal-coordinate analysis of (A) the Bray-Curtis dissimilarity matrix and (B) weighted Unifrac distances. The results shown were analyzed following the QIIME 1 pipeline and Greengenes database. Download FIG S5, EPS file, 2.4 MB. Copyright © 2018 Xue et al. 2018 Xue et al. This content is distributed under the terms of the Creative Commons Attribution 4.0 International license.

**FIG 4:**
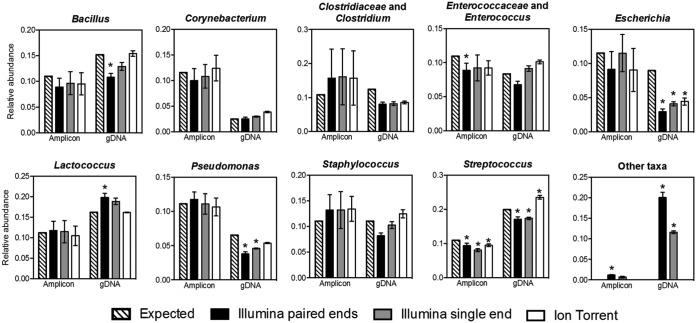
Relative abundance of taxa in the 16S rRNA PCR amplicon and gDNA mock communities. Relative abundances of expected taxa are labeled with the corresponding taxonomic level from sequencing results. “Amplicon” represents the 16S rRNA PCR amplicon mock community, and “gDNA” represents the gDNA mock community. The results shown were analyzed following the DADA2 pipeline with the Greengenes database. Each bar represents the mean ± SD from three replicates. Proportions for each community were compared to expected proportions using ANOVA with Bonferroni’s multiple-comparison test. *P* values of <0.05 were considered to be significantly different from the expected values and are indicated by an asterisk above each bar plot.

Examination of the relative abundances of individual taxa across the three DNA sequencing approaches showed that for the 16S rRNA PCR amplicon mock community, the proportions of most bacterial species were mostly not significantly altered compared to expected theoretical values. Exceptions to this finding were the reduced proportions of Enterococcaceae and Enterococcus found for Illumina paired-end assemblies and Streptococcus for both Illumina MiSeq methods as well as the Ion Torrent PGM platform (Fig. 4). For the gDNA mock community, the proportions of Escherichia and Streptococcus were significantly different from expected for all three DNA sequencing platforms. The proportions of Pseudomonas were also significantly lower than expected for the single- and paired-end assemblies from the Illumina MiSeq, and the proportions of Bacillus and Lactococcus were also altered for the paired-end assemblies. Lastly, there were higher proportions of “other” taxa for both Illumina MiSeq methods (Fig. 4), especially in the gDNA mock community. Overall, even though DNA sequencing with the Ion Torrent PGM combined with DADA2/Greengenes analysis did not completely provide the expected bacterial composition, this approach resulted in the most accurate representations of the bacteria and their proportions in both gDNA and PCR amplicon mock communities.

## DISCUSSION

By comparing DNA sequencing methods, analysis algorithms, and reference databases using dairy relevant bacterial DNA (PCR amplicon and gDNA) mock communities, we found that the DADA2/Greengenes data analysis methods with the Ion Torrent PGM yielded the most accurate interpretations of the 16S rRNA V4 variable region relative to the other methods (Illumina MiSeq, QIIME 1, RDP) tested. This conclusion is notable considering that DADA2 was developed for analysis of Illumina DNA sequence reads (26). Although successfully applied for that purpose (25, 33), our findings show that the DADA2 algorithm is compatible with the Ion Torrent reads and error profile. Moreover, our study also offers new and detailed 16S rRNA data comparisons on single- versus multitemplate PCR and single- versus pair-end assembled Illumina reads, which can be broadly informative to benchmark bioinformatics workflows and to the study of bacterial diversity and composition in other microbial habitats besides dairy products.

Application of DADA2 and QIIME 1 (UCLUST) analysis pipelines to the same 16S rRNA gene data showed that DADA2 assigned fewer total and spurious OTUs/ASVs than QIIME 1 even with stringent filtering (32). Because read length was kept consistent within each DNA sequencing and data assembly platform, platform-specific differences in OTU/ASV numbers were mainly derived from the core algorithms used for filtering and clustering representative sequences. The DADA2 core algorithm includes error-rate-based denoising, isBimeraDenovo chimera identification, and ASV inference (26). In QIIME 1, the core analysis includes the USEARCH chimera identification and OTU picking strategy (34). Comparison of OTUs and ASVs using the QIIME 1 and DADA2 pipelines, respectively, also showed that the DADA2 analysis pipeline was able to assign ASVs to more specific taxonomic levels (genus) than QIIME 1. This could be the result of the different taxonomy classifiers employed by DADA2 (RDP’s naive Bayesian classifier) and QIIME 1 (UCLUST classifier) (35).

OTU and ASV taxonomy assignments were also compared with consideration to 16S rRNA gene reference databases. Results from the different combinations of analysis methods and reference databases showed that the majority of OTUs/ASVs detected were representatives of bacterial taxa included in the 16S rRNA PCR amplicon and gDNA mock communities. Each bacterial species was represented by at least a single OTU/ASV. Additional OTUs/ASVs were largely due to low-abundance sequence variants. DNA sequences of the predominant ASVs/OTUs were 100% identical between gDNA and PCR amplicon mock communities, further supporting the precision of the technique. When QIIME 1 was applied, the RDP_GOLD database (36) yielded lower numbers of total OTUs than found with Greengenes 13.8, independent of whether the Illumina MiSeq or Ion Torrent PGM was used to generate the DNA sequence reads. However, the RDP_GOLD database has not been updated since 2011 (36) and could potentially be missing many bacterial sequences, leading to less differentiation between OTUs. With the DADA2 pipeline, ASVs were inferred prior to taxonomy assignment (26), resulting in the same total ASV numbers for both the RDP 11.5 and Greengenes 13.8 databases. However, assignments of DADA2 ASVs were still influenced by reference database-specific taxonomic nomenclature and DNA sequences (37), such that Greengenes provided deeper, more accurate taxonomic assignments than those found with RDP.

The Illumina MiSeq and Ion Torrent PGM methods also clearly impacted the outcomes of our mock community analyses. The Illumina MiSeq is well established and known for its low error rate, high-volume read outputs, and low sequencing cost per Gb (38, 39). Although Illumina MiSeq reads had higher Phred quality scores, for both single-end and paired-end assembled Illumina MiSeq reads, greater numbers of unexpected taxa and OTUs/ASVs were observed compared to the Ion Torrent PGM. This finding could be the result of differences in library preparation methods, external contamination, index switching (40), and/or substitution errors (41, 42) specific to the Illumina MiSeq. To reduce misassigned reads, previous studies have suggested using a dual-index strategy (43) and stringent filtering at the index region (40), as well as sequencing of negative controls for *in silico* removal of contaminant reads (44). In contrast, the use of the Ion Torrent PGM with our read trimming parameters resulted in the lowest numbers of DADA2 assigned ASVs. At 13 ASVs for both mock communities, this number was only slightly greater than the nine predicted. All 13 ASVs were repeatedly assigned to members of the mock communities, except for one low-abundance ASV when RDP was applied. The four additional ASVs were the result of read errors in the homopolymer regions, a common Ion Torrent error model (20, 38, 39) that still passed the DADA2 filtering with recommended parameters (https://benjjneb.github.io/dada2/faq.html#can-i-use-dada2-with-my-454-or-ion-torrent-data). This error model could be further reduced by increasing the homopolymer error penalty value. Interestingly, the Ion Torrent PGM reads resulted in the highest numbers of OTUs when QIIME 1 was used to analyze the data. This might have been due to the higher number of erroneous reads that were passed by QIIME 1 filtering, but were identified as sequence chimeras and artifacts by DADA2.

For the PCR amplicon mock community, bacterial diversity analyses based on the DADA2/Greengenes pipeline showed that the results from the Illumina MiSeq were similar to Ion Torrent PGM and the *in silico* expected values. However, the gDNA mock community relative abundances of certain bacteria in the gDNA mock community were significantly altered compared to expected proportions according to the paired- and single-end Illumina MiSeq methods. This was particularly the case for Streptococcus and Lactococcus. Because Streptococcus and Lactococcus have similar 16S rRNA gene sequences, variation in their relative abundances could be caused by the accumulation of substitution errors, a common error that occurs with the Illumina MiSeq instrument (41, 42). No spurious taxa were found in either the PCR amplicon or gDNA mock community in Ion Torrent results analyzed with the DADA2/Greengenes pipeline. In contrast, the gDNA mock community contained 9-fold and 5-fold more “other” spurious taxa compared to the PCR amplicon mock community in Illumina paired- and single-end results, respectively. To this regard, the majority of these taxa (and proportion of reads, >74%) were assigned to bacterial orders, families, and genera that are highly related to the species included in the mock community (e.g., Clostridiales, Lactobacillus, Oscillospira, and Turicibacter). This, together with the lower numbers of ASVs found for the corresponding PCR amplicon community and single-end results, indicates that errors resulting from paired-end Illumina MiSeq assembly are augmented by combining multitemplate PCR with joining forward and reverse reads. This issue can be mitigated by using single-end reads (as shown by the data here), fewer PCR cycles (33), and increasing the denaturing time (45).

By the use of bacterial DNA standards from nine dairy-relevant bacterial species, we found that DNA sequencing and analysis pipelines contributed significant variations to OTU/ASV distributions and observed bacterial diversities. Moreover, PCR biases and errors from multitemplate DNA amplifications are not entirely filtered with the Illumina MiSeq method. Overall, the Ion Torrent PGM DNA sequencer combined with the DADA2/Greengenes pipeline led to more accurate OTU/ASV assignments and bacterial diversity measurements of the PCR amplicon and gDNA mock communities under our study conditions. The Ion Torrent PGM method is recognized for shorter run times, lower instrument cost, and flexibility in sequencing scale per run by the use of different sequencing chips (38, 39). Therefore, this platform could be of particular use to study dairy and other food products with short shelf life times. Moreover, with DADA2 being wrapped in the QIIME 2 platform, we agree with the QIIME 2 developers that new sequencing results should be analyzed using QIIME 2 with a standardized analysis pipeline (e.g., DADA2) instead of QIIME 1 (UCLUST) (46). Further improvements might be reached by refinements to taxonomy classifiers (35), updating reference databases to emphasize bacteria found in different environments, such as dairy foods, and/or testing other reference databases, such as SILVA (37, 47, 48). Lastly, we recognize that upstream sample processing and DNA extraction protocols can introduce significant biases into assessments of bacterial community composition (1–15). Therefore, the data analysis methods applied here should be tested using whole-cell mock communities containing different proportions of bacteria as well as on complex environmental samples. Moreover, to increase reproducibility, consistent methodology and inclusion of negative and positive controls in each run/project are recommended (49). The findings here and the continued development of microbial diversity analysis methods should result in even more reliable comparisons within and between bacterial habitats.

## MATERIALS AND METHODS

### Bacterial strains and culture conditions.

Bacterial strains representing species commonly found in bovine milk were used to construct a mock bacterial community (Table 1). Each bacterial strain was grown in standard laboratory culture medium with negative controls for that species and harvested at early stationary phase by centrifugation at 13,000 × *g* for 2 min. The laboratory culture media were as follows: Bacillus subtilis, Pseudomonas fluorescens, and Escherichia coli, LB (Lennox broth; Thermo Fisher Scientific); Enterococcus faecalis and Streptococcus agalactiae, brain heart infusion broth (Thermo Fisher Scientific); Staphylococcus aureus, tryptic soy broth (Becton Dickinson); Corynebacterium bovis, tryptic soy broth (Becton Dickinson) with 0.1% Tween 80; Lactococcus lactis, M17 broth (Becton Dickinson) with 0.5% glucose; and Clostridium tyrobutyricum, reinforced clostridial broth (Becton Dickinson). All strains were incubated at 37°C, with the exception of B. subtilis, L. lactis, and P. fluorescens, which were incubated at 30°C. B. subtilis, C. bovis, E. faecalis, E. coli, and P. fluorescens were grown under aeration (250 rpm).

### Genomic DNA extraction and PCR amplification.

Genomic DNA was extracted using the MagMAX Total nucleic acid isolation kit (Thermo Fisher Scientific, Vilnius, Lithuania) according to the manufacturer’s protocol with the repeat bead beating method on a FastPrep-24 instrument (MP Biomedicals LLC). The DNA concentration was measured with the Qubit 3.0 fluorometer using the Qubit double-stranded DNA (dsDNA) HS assay kit (Life Technologies, Eugene, OR). PCR amplification was performed using *Ex Taq* DNA polymerase (TaKaRa, Otsu, Japan) and primers F515 and R806 (50) with a random 8-bp barcode on the 5ʹ end of F515 for sample multiplexing (51, 52). PCR was initiated at 94°C for 3 min, followed by 35 cycles of 94°C for 45 s, 54°C for 60 s, and 72°C for 30 s, with a final extension step at 72°C for 10 min. Negative controls were run for each barcoded primer. No PCR product for the negative controls was observed on a 1.5% agarose gel. PCR products were pooled and then gel purified with the Wizard SV gel and PCR clean-up system (Promega, Madison, WI).

### Preparation of the mock communities.

A schematic experimental design for preparing the mock communities is shown in Fig. 1. For the gDNA mock community, 100 ng gDNA isolated from each of the strains was pooled in three separate replicates. The proportion of each bacterial strain in the gDNA mock community was determined by taking into account the genome size and 16S rRNA gene copy number (Table 1). To construct the amplicon mock community, gDNA of the nine bacterial strains was amplified in triplicate by using three different barcoded PCR primers. Amplicon concentrations were measured with the Quant-iT PicoGreen dsDNA assay kit (Life Technologies, Eugene, OR) prior to pooling at equal molar concentrations.

### DNA sequencing.

For Illumina sequencing, the KAPA HTP library preparation kit (KK8234, Kapa Biosystems, Pittsburgh, PA) was used for the ligation of NEXTflex adapters (Bioo Scientific, Austin, TX) to the 16S rRNA amplicons prior to 250-bp paired-end sequencing (with 7% PhiX control) on an Illumina MiSeq instrument at the University of California, Davis, Genome Center (http://genomecenter.ucdavis.edu/). For Ion Torrent sequencing, non-barcoded Ion A and Ion P1 adapters were ligated to the pooled amplicons, followed by templating, enrichment, and sequencing on the One-Touch 2 and One-Touch ES systems and Ion PGM using the 400 sequencing kit and a 318 v2 chip (Life Technologies, Carlsbad, CA).

### 16S rRNA gene sequence analysis.

An *in silico* mock community, termed “expected,” was created using the 16S V4 amplicon sequences from published genomes and reference genomes for the specific bacterial species (Table 1). In addition, the expected 16S V4 region copy numbers were normalized based on the genome size and 16S rRNA gene copy numbers.

Illumina MiSeq sequencing outputs were trimmed with the fastx_tools (53) to keep the first 245 and 170 bases for the forward and reverse reads, respectively (for quality profiles, see Fig. S6 and S7 in the supplemental material). The Ion Torrent sequence output BAM file was converted to FASTQ format using BEDTools (54), and reads shorter than 200 bp were also removed. The first 280 bases of the Ion Torrent reads were kept for analysis (for quality profiles, see Fig. S6 and S7 in the supplemental material).

10.1128/mSphere.00410-18.6 FIG S6 Quality profiles of all (A) Illumina MiSeq forward reads, (B) Illumina MiSeq reverse reads, and (C) Ion Torrent PGM reads. Download FIG S6, TIF file, 2.1 MB. Copyright © 2018 Xue et al. 2018 Xue et al. This content is distributed under the terms of the Creative Commons Attribution 4.0 International license.

10.1128/mSphere.00410-18.7 FIG S7 Quality profile of trimmed (A) Illumina MiSeq forward reads, (B) Illumina MiSeq reverse reads, and (C) Ion Torrent PGM reads. Download FIG S7, TIF file, 2.1 MB. Copyright © 2018 Xue et al. 2018 Xue et al. This content is distributed under the terms of the Creative Commons Attribution 4.0 International license.

The FASTQ files were then analyzed with QIIME version 1.9.1 and DADA2 1.6.0 (26, 55). In QIIME 1, Illumina reads from the two orientations (forward and reverse) were analyzed either with or without assembly where the *join_paired_ends.py* (*fastq-join* method) (56) script was used with minimum 100-bp overlap and 1% maximum difference between overlapping sequences. Ion Torrent single-end and paired-end assembled Illumina FASTQ files then had the barcode (8 bases) and primer regions (forward primer, 21 bases; reverse primer, 20 bases) removed and were demultiplexed using the *split_libraries_fastq.py* script with no barcode error and quality filtered at Q30. Chimeric sequences were identified using USEARCH (34, 36) with both the *de novo* and reference-based methods against the Greengene database version 13.8 (57, 58) via the *identify_chimeric_seqs.py* command with default parameter values. Sequences from both Illumina and Ion Torrent as well as the *in silico* mock community with expected proportions were merged as one fasta file for operational taxonomic unit (OTU) clustering using the *pick_open_reference_otus.py* script with recommended parameters (32) and the UCLUST method at 97% similarity thresholds. The Greengenes version 13.8 (57, 58) and RDP_GOLD (36) databases were used as references for OTU assignments. Archaea, chloroplasts, and low-abundance (0.005%) OTUs were removed from the OTU tables (32).

In DADA2, for single-end analysis, the truncated Illumina and Ion Torrent FASTQ files after barcode (8 bases) and primer sequence (forward primer, 21 bases; reverse primer, 20 bases) trimming were demultiplexed using *split_libraries_fastq.py* script with no barcode error and no quality filter (-r 999, -n 999, -q 0, -p 0.0001). Since the single-end reads were already quality trimmed, no additional truncation was performed in DADA2 to be consistent in read length with QIIME 1 analysis. For paired-end analysis, in order to get matched sequence files, raw Illumina reads were demultiplexed in pairs using the idemp tool (59) with no barcode error. Barcode and forward and reverse primer regions were then trimmed with fastx_tools (53). The resulting reads were truncated in DADA2 to keep the first 196 bases of the forward reads and 121 bases of the reverse reads, which were later merged after ASV inference with no error allowed and a 51-bp minimum overlap to be consistent with the QIIME 1 method in resulting read length. For reads from both Ion Torrent and Illumina MiSeq, the error model learning [*learnErrors()*], dereplication [*derepFastq()*], and ASV inference [*dada()*] were performed in R with the DADA2 default parameter, except for added parameters for Ion Torrent [*dada(HOMOPOLYMER_GAP_PENALTY=-1, BAND_SIZE = 32)*]. Chimeras were identified and removed after sequence clustering via the *removeBimeraDenovo()* function with the “consensus” method and the *isBimeraDenovoTable()* function default settings.

Taxonomy was assigned to the resulting amplicon sequence variants (ASVs) using RDP database version 11.5 (60) and Greengenes database version 13.8 with the minimum bootstrap confidence at 80 (57, 58). Ion Torrent and Illumina single-end and paired-end assembled reads were merged with the *in silico* mock community using the phyloseq package in R (61), and singletons and low-abundance (0.005%) ASVs were removed to be consistent with QIIME 1 analysis. Sequences of spurious ASVs were further aligned with sequences in the NCBI nr/nt database using BLASTn (62) with default settings.

### Statistics.

OTU/ASV counts were rarefied at 5,483 sequences per sample to retain all samples for downstream analyses. Significant differences in the observed mock community composition (α diversity and taxonomic distribution) were determined by analysis of variance (ANOVA) with the Bonferroni’s multiple-comparison test. A *P* value of <0.05 indicates significance. The significance of sample clustering was indicated by permutational multivariate ANOVA using the adonis function from the vegan package in R (63) with a *P* value of <0.05 through 9,999 permutations.

### Accession number(s).

Joined- and single-end DNA sequences after quality filtering and trimming have been deposited in the Qiita database (https://qiita.ucsd.edu) under study ID no. 11351 and in the European Nucleotide Archive (ENA) under accession no. ERP104377.

## References

[1] Dominianni C, Wu J, Hayes RB, Ahn J 2014 Comparison of methods for fecal microbiome biospecimen collection. BMC Microbiol 14:103. doi:10.1186/1471-2180-14-103. 24758293 PMC4005852

[2] Flores R, Shi JX, Yu GQ, Ma B, Ravel J, Goedert JJ, Sinha R 2015 Collection media and delayed freezing effects on microbial composition of human stool. Microbiome 3:33. doi:10.1186/s40168-015-0092-7. 26269741 PMC4534027

[3] Hsieh YH, Peterson CM, Raggio A, Keenan MJ, Martin RJ, Ravussin E, Marco ML 2016 Impact of different fecal processing methods on assessments of bacterial diversity in the human intestine. Front Microbiol 7:1643. doi:10.3389/fmicb.2016.01643. 27812352 PMC5071325

[4] Fouhy F, Clooney AG, Stanton C, Claesson MJ, Cotter PD 2016 16S rRNA gene sequencing of mock microbial populations—impact of DNA extraction method, primer choice and sequencing platform. BMC Microbiol 16:123. doi:10.1186/s12866-016-0738-z. 27342980 PMC4921037

[5] Fouhy F, Deane J, Rea MC, O’Sullivan Ó, Ross RP, O’Callaghan G, Plant BJ, Stanton C 2015 The effects of freezing on faecal microbiota as determined using MiSeq sequencing and culture-based investigations. PLoS One 10:e0119355. doi:10.1371/journal.pone.0119355. 25748176 PMC4352061

[6] Rubin BER, Gibbons SM, Kennedy S, Hampton-Marcell J, Owens S, Gilbert JA 2013 Investigating the impact of storage conditions on microbial community composition in soil samples. PLoS One 8:e70460. doi:10.1371/journal.pone.0070460. 23936206 PMC3729949

[7] Wu GD, Lewis JD, Hoffmann C, Chen YY, Knight R, Bittinger K, Hwang J, Chen J, Berkowsky R, Nessel L, Li HZ, Bushman FD 2010 Sampling and pyrosequencing methods for characterizing bacterial communities in the human gut using 16S sequence tags. BMC Microbiol 10:206. doi:10.1186/1471-2180-10-206. 20673359 PMC2921404

[8] Carroll IM, Ringel-Kulka T, Siddle JP, Klaenhammer TR, Ringel Y 2012 Characterization of the fecal microbiota using high-throughput sequencing reveals a stable microbial community during storage. PLoS One 7:e46953. doi:10.1371/journal.pone.0046953. 23071673 PMC3465312

[9] Bai GY, Gajer P, Nandy M, Ma B, Yang HQ, Sakamoto J, Blanchard MH, Ravel J, Brotman RM 2012 Comparison of storage conditions for human vaginal microbiome studies. PLoS One 7:e36934. doi:10.1371/journal.pone.0036934. 22655031 PMC3360033

[10] Quigley L, O'Sullivan O, Beresford TP, Ross RP, Fitzgerald GF, Cotter PD 2012 A comparison of methods used to extract bacterial DNA from raw milk and raw milk cheese. J Appl Microbiol 113:96–105. doi:10.1111/j.1365-2672.2012.05294.x. 22452460

[11] Barratt MJ, Lebrilla C, Shapiro HY, Gordon JI 2017 The gut microbiota, food science, and human nutrition: a timely marriage. Cell Host Microbe 22:134–141. doi:10.1016/j.chom.2017.07.006. 28799899 PMC5915309

[12] Mackenzie BW, Waite DW, Taylor MW 2015 Evaluating variation in human gut microbiota profiles due to DNA extraction method and inter-subject differences. Front Microbiol 6:130. doi:10.3389/fmicb.2015.00130. 25741335 PMC4332372

[13] Salter SJ, Cox MJ, Turek EM, Calus ST, Cookson WO, Moffatt MF, Turner P, Parkhill J, Loman NJ, Walker AW 2014 Reagent and laboratory contamination can critically impact sequence-based microbiome analyses. BMC Biol 12:87. doi:10.1186/s12915-014-0087-z. 25387460 PMC4228153

[14] Yuan SQ, Cohen DB, Ravel J, Abdo Z, Forney LJ 2012 Evaluation of methods for the extraction and purification of DNA from the human microbiome. PLoS One 7:e33865. doi:10.1371/journal.pone.0033865. 22457796 PMC3311548

[15] Kennedy NA, Walker AW, Berry SH, Duncan SH, Farquarson FM, Louis P, Thomson JM, Satsangi J, Flint HJ, Parkhill J, Lees CW, Hold GL, UK IBD Genetics Consortium 2014 The impact of different DNA extraction kits and laboratories upon the assessment of human gut microbiota composition by 16S rRNA gene sequencing. PLoS One 9:e88982. doi:10.1371/journal.pone.0088982. 24586470 PMC3933346

[16] Brandariz-Fontes C, Camacho-Sanchez M, Vilà C, Vega-Pla JL, Rico C, Leonard JA 2015 Effect of the enzyme and PCR conditions on the quality of high-throughput DNA sequencing results. Sci Rep 5:8056. doi:10.1038/srep08056. 25623996 PMC4306961

[17] Gonzalez JM, Portillo MC, Belda-Ferre P, Mira A 2012 Amplification by PCR artificially reduces the proportion of the rare biosphere in microbial communities. PLoS One 7:e29973. doi:10.1371/journal.pone.0029973. 22253843 PMC3256211

[18] Tremblay J, Singh K, Fern A, Kirton ES, He S, Woyke T, Lee J, Chen F, Dangl JL, Tringe SG 2015 Primer and platform effects on 16S rRNA tag sequencing. Front Microbiol 6:771. doi:10.3389/fmicb.2015.00771. 26300854 PMC4523815

[19] D’Amore R, Ijaz UZ, Schirmer M, Kenny JG, Gregory R, Darby AC, Shakya M, Podar M, Quince C, Hall N 2016 A comprehensive benchmarking study of protocols and sequencing platforms for 16S rRNA community profiling. BMC Genomics 17:55. doi:10.1186/s12864-015-2194-9. 26763898 PMC4712552

[20] Salipante SJ, Kawashima T, Rosenthal C, Hoogestraat DR, Cummings LA, Sengupta DJ, Harkins TT, Cookson BT, Hoffman NG 2014 Performance comparison of Illumina and Ion Torrent next-generation sequencing platforms for 16S rRNA-based bacterial community profiling. Appl Environ Microbiol 80:7583–7591. doi:10.1128/AEM.02206-14. 25261520 PMC4249215

[21] Nelson MC, Morrison HG, Benjamino J, Grim SL, Graf J 2014 Analysis, optimization and verification of Illumina-generated 16S rRNA gene amplicon surveys. PLoS One 9:e94249. doi:10.1371/journal.pone.0094249. 24722003 PMC3983156

[22] Sinclair L, Osman OA, Bertilsson S, Eiler A 2015 Microbial community composition and diversity via 16S rRNA gene amplicons: evaluating the Illumina platform. PLoS One 10:e0116955. doi:10.1371/journal.pone.0116955. 25647581 PMC4315398

[23] Golob JL, Margolis E, Hoffman NG, Fredricks DN 2017 Evaluating the accuracy of amplicon-based microbiome computational pipelines on simulated human gut microbial communities. BMC Bioinformatics 18:283. doi:10.1186/s12859-017-1690-0. 28558684 PMC5450146

[24] Kopylova E, Navas-Molina JA, Mercier C, Xu ZZ, Mahe F, He Y, Zhou HW, Rognes T, Caporaso JG, Knight R 2016 Open-source sequence clustering methods improve the state of the art. mSystems 1:e00003-15. doi:10.1128/mSystems.00003-15. PMC5069751 27822515

[25] Amir A, McDonald D, Navas-Molina JA, Kopylova E, Morton JT, Zech Xu Z, Kightley EP, Thompson LR, Hyde ER, Gonzalez A, Knight R 2017 Deblur rapidly resolves single-nucleotide community sequence patterns. mSystems 2:e00191-16. doi:10.1128/mSystems.00191-16. 28289731 PMC5340863

[26] Callahan BJ, McMurdie PJ, Rosen MJ, Han AW, Johnson AJ, Holmes SP 2016 DADA2: high-resolution sample inference from Illumina amplicon data. Nat Methods 13:581. doi:10.1038/nmeth.3869. 27214047 PMC4927377

[27] Edgar RC 2017 Accuracy of microbial community diversity estimated by closed- and open-reference OTUs. PeerJ 5:e3889. doi:10.7717/peerj.3889. 29018622 PMC5631090

[28] Westcott SL, Schloss PD 2015 *De novo* clustering methods outperform reference-based methods for assigning 16S rRNA gene sequences to operational taxonomic units. PeerJ 3:e1487. doi:10.7717/peerj.1487. 26664811 PMC4675110

[29] He Y, Caporaso JG, Jiang XT, Sheng HF, Huse SM, Rideout JR, Edgar RC, Kopylova E, Walters WA, Knight R, Zhou HW 2015 Stability of operational taxonomic units: an important but neglected property for analyzing microbial diversity. Microbiome 3:20. doi:10.1186/s40168-015-0081-x. 25995836 PMC4438525

[30] Callahan BJ, McMurdie PJ, Holmes SP 2017 Exact sequence variants should replace operational taxonomic units in marker-gene data analysis. ISME J 11:2639. doi:10.1038/ismej.2017.119. 28731476 PMC5702726

[31] Edgar RC 2018 Updating the 97% identity threshold for 16S ribosomal RNA OTUs. Bioinformatics 34:2371–2375. 29506021 10.1093/bioinformatics/bty113

[32] Bokulich NA, Subramanian S, Faith JJ, Gevers D, Gordon JI, Knight R, Mills DA, Caporaso JG 2013 Quality-filtering vastly improves diversity estimates from Illumina amplicon sequencing. Nat Methods 10:57–59. doi:10.1038/nmeth.2276. 23202435 PMC3531572

[33] Allali I, Arnold JW, Roach J, Cadenas MB, Butz N, Hassan HM, Koci M, Ballou A, Mendoza M, Ali R, Azcarate-Peril MA 2017 A comparison of sequencing platforms and bioinformatics pipelines for compositional analysis of the gut microbiome. BMC Microbiol 17:194. doi:10.1186/s12866-017-1101-8. 28903732 PMC5598039

[34] Edgar RC 2010 Search and clustering orders of magnitude faster than BLAST. Bioinformatics 26:2460–2461. doi:10.1093/bioinformatics/btq461. 20709691

[35] Bokulich NA, Kaehler BD, Rideout JR, Dillon M, Bolyen E, Knight R, Huttley GA, Caporaso JG 2018 Optimizing taxonomic classification of marker gene amplicon sequences. PeerJ Preprints. doi:10.7287/peerj.preprints.3208v2. PMC5956843 29773078

[36] Edgar RC, Haas BJ, Clemente JC, Quince C, Knight R 2011 UCHIME improves sensitivity and speed of chimera detection. Bioinformatics 27:2194–2200. doi:10.1093/bioinformatics/btr381. 21700674 PMC3150044

[37] Balvočiūtė M, Huson DH 2017 SILVA, RDP, Greengenes, NCBI and OTT—how do these taxonomies compare? BMC Genomics 18:114. doi:10.1186/s12864-017-3501-4. 28361695 PMC5374703

[38] Quail MA, Smith M, Coupland P, Otto TD, Harris SR, Connor TR, Bertoni A, Swerdlow HP, Gu Y 2012 A tale of three next generation sequencing platforms: comparison of Ion Torrent, Pacific Biosciences and Illumina MiSeq sequencers. BMC Genomics 13:341. doi:10.1186/1471-2164-13-341. 22827831 PMC3431227

[39] Goodwin S, McPherson JD, McCombie WR 2016 Coming of age: ten years of next-generation sequencing technologies. Nat Rev Genet 17:333. doi:10.1038/nrg.2016.49. 27184599 PMC10373632

[40] Wright ES, Vetsigian KH 2016 Quality filtering of Illumina index reads mitigates sample cross-talk. BMC Genomics 17:876. doi:10.1186/s12864-016-3217-x. 27814679 PMC5097354

[41] Schirmer M, D’Amore R, Ijaz UZ, Hall N, Quince C 2016 Illumina error profiles: resolving fine-scale variation in metagenomic sequencing data. BMC Bioinformatics 17:125. doi:10.1186/s12859-016-0976-y. 26968756 PMC4787001

[42] Schirmer M, Ijaz UZ, D'Amore R, Hall N, Sloan WT, Quince C 2015 Insight into biases and sequencing errors for amplicon sequencing with the Illumina MiSeq platform. Nucleic Acids Res 43:e37. doi:10.1093/nar/gku1341. 25586220 PMC4381044

[43] Kircher M, Sawyer S, Meyer M 2012 Double indexing overcomes inaccuracies in multiplex sequencing on the Illumina platform. Nucleic Acids Res 40:e3. doi:10.1093/nar/gkr771. 22021376 PMC3245947

[44] Davis NM, Proctor D, Holmes SP, Relman DA, Callahan BJ 2017 Simple statistical identification and removal of contaminant sequences in marker-gene and metagenomics data. bioRxiv doi:10.1101/221499. PMC6298009 30558668

[45] Laursen MF, Dalgaard MD, Bahl MI 2017 Genomic GC-content affects the accuracy of 16S rRNA gene sequencing based microbial profiling due to PCR bias. Front Microbiol 8:1934. doi:10.3389/fmicb.2017.01934. 29051756 PMC5633598

[46] Caporaso G 2017 A response to “Accuracy of microbial community diversity …” by R Edgar for QIIME users. https://forum.qiime2.org/t/a-response-to-accuracy-of-microbial-community-diversity-by-r-edgar-for-qiime-users/1456. Accessed October 2017.

[47] Quast C, Pruesse E, Yilmaz P, Gerken J, Schweer T, Yarza P, Peplies J, Glockner FO 2012 The SILVA ribosomal RNA gene database project: improved data processing and web-based tools. Nucleic Acids Res 41:D590–D596. doi:10.1093/nar/gks1219. 23193283 PMC3531112

[48] Yilmaz P, Parfrey LW, Yarza P, Gerken J, Pruesse E, Quast C, Schweer T, Peplies J, Ludwig W, Glockner FO 2014 The SILVA and "All-species Living Tree Project (LTP)" taxonomic frameworks. Nucleic Acids Res 42:D643–D648. doi:10.1093/nar/gkt1209. 24293649 PMC3965112

[49] Pollock J, Glendinning L, Wisedchanwet T, Watson M 2018 The madness of microbiome: attempting to find consensus “best practice” for 16S microbiome studies. Appl Environ Microbiol 84:e02627-17. doi:10.1128/AEM.02627-17. 29427429 PMC5861821

[50] Caporaso JG, Lauber CL, Walters WA, Berg-Lyons D, Lozupone CA, Turnbaugh PJ, Fierer N, Knight R 2011 Global patterns of 16S rRNA diversity at a depth of millions of sequences per sample. Proc Natl Acad Sci U S A 108:4516–4522. doi:10.1073/pnas.1000080107. 20534432 PMC3063599

[51] Bokulich NA, Joseph CML, Allen G, Benson AK, Mills DA 2012 Next-generation sequencing reveals significant bacterial diversity of botrytized wine. PLoS One 7:e36357. doi:10.1371/journal.pone.0036357. 22563494 PMC3341366

[52] Hamady M, Walker JJ, Harris JK, Gold NJ, Knight R 2008 Error-correcting barcoded primers for pyrosequencing hundreds of samples in multiplex. Nat Methods 5:235–237. doi:10.1038/nmeth.1184. 18264105 PMC3439997

[53] Hannon Lab. 2014 FASTX-Toolkit: FASTQ/A short-reads pre-processing tools. http://hannonlab.cshl.edu/fastx_toolkit/download.html. Accessed 30 May 2017.

[54] Quinlan AR, Hall IM 2010 BEDTools: a flexible suite of utilities for comparing genomic features. Bioinformatics 26:841–842. doi:10.1093/bioinformatics/btq033. 20110278 PMC2832824

[55] Caporaso JG, Kuczynski J, Stombaugh J, Bittinger K, Bushman FD, Costello EK, Fierer N, Peña AG, Goodrich JK, Gordon JI, Huttley GA, Kelley ST, Knights D, Koenig JE, Ley RE, Lozupone CA, McDonald D, Muegge BD, Pirrung M, Reeder J, Sevinsky JR, Turnbaugh PJ, Walters WA, Widmann J, Yatsunenko T, Zaneveld J, Knight R 2010 QIIME allows analysis of high-throughput community sequencing data. Nat Methods 7:335–336. doi:10.1038/nmeth.f.303. 20383131 PMC3156573

[56] Aronesty E 2013 Comparison of sequencing utility programs. Open Bioinformatics J 7:1. doi:10.2174/1875036201307010001.

[57] McDonald D, Price MN, Goodrich J, Nawrocki EP, DeSantis TZ, Probst A, Andersen GL, Knight R, Hugenholtz P 2012 An improved Greengenes taxonomy with explicit ranks for ecological and evolutionary analyses of bacteria and archaea. ISME J 6:610–618. doi:10.1038/ismej.2011.139. 22134646 PMC3280142

[58] Werner JJ, Koren O, Hugenholtz P, DeSantis TZ, Walters WA, Caporaso JG, Angenent LT, Knight R, Ley RE 2012 Impact of training sets on classification of high-throughput bacterial 16S rRNA gene surveys. ISME J 6:94–103. doi:10.1038/ismej.2011.82. 21716311 PMC3217155

[59] Wu Y 2014 Barcode demultiplex for Illumina I1, R1, R2 fastq.gz files. https://github.com/yhwu/idemp. Accessed 20 November 2017.

[60] Cole JR, Wang Q, Fish JA, Chai B, McGarrell DM, Sun Y, Brown CT, Porras-Alfaro A, Kuske CR, Tiedje JM 2014 Ribosomal Database Project: data and tools for high throughput rRNA analysis. Nucleic Acids Res 42:D633–D642. doi:10.1093/nar/gkt1244. 24288368 PMC3965039

[61] McMurdie PJ, Holmes S 2013 phyloseq: an R package for reproducible interactive analysis and graphics of microbiome census data. PLoS One 8:e61217. doi:10.1371/journal.pone.0061217. 23630581 PMC3632530

[62] Altschul SF, Gish W, Miller W, Myers EW, Lipman DJ 1990 Basic local alignment search tool. J Mol Biol 215:403–410. doi:10.1016/S0022-2836(05)80360-2. 2231712

[63] Oksanen J, Friendly M, Kindt R, Legendre P, McGlinn D, Minchin PR, O'Hara RB, Simpson GL, Solymos P, Stevens MHM, Szoecs E, Wagner H 2018 vegan: community ecology package. https://cran.r-project.org/web/packages/vegan/index.html. Accessed 21 February 2018.

[64] Lee J, Jang YS, Han MJ, Kim JY, Lee SY 2016 Deciphering *Clostridium tyrobutyricum* metabolism based on the whole-genome sequence and proteome analyses. mBio 7:e00743-16. doi:10.1128/mBio.00743-16. 27302759 PMC4916380

[65] Schroder J, Glaub A, Schneider J, Trost E, Tauch A 2012 Draft genome sequence of *Corynebacterium bovis* DSM 20582, which causes clinical mastitis in dairy cows. J Bacteriol 194:4437. doi:10.1128/JB.00839-12. 22843578 PMC3416247

[66] Kim EB, Kopit LM, Harris LJ, Marco ML 2012 Draft genome sequence of the quality control strain *Enterococcus faecalis* ATCC 29212. J Bacteriol 194:6006–6007. doi:10.1128/JB.01423-12. 23045510 PMC3486070

[67] Bolotin A, Wincker P, Mauger S, Jaillon O, Malarme K, Weissenbach J, Ehrlich SD, Sorokin A 2001 The complete genome sequence of the lactic acid bacterium *Lactococcus lactis* ssp *lactis* IL1403. Genome Res 11:731–753. doi:10.1101/gr.169701. 11337471 PMC311110

[68] Loper JE, Hassan KA, Mavrodi DV, Davis EW, Lim CK, Shaffer BT, Elbourne LDH, Stockwell VO, Hartney SL, Breakwell K, Henkels MD, Tetu SG, Rangel LI, Kidarsa TA, Wilson NL, de Mortel JEV, Song CX, Blumhagen R, Radune D, Hostetler JB, Brinkac LM, Durkin AS, Kluepfel DA, Wechter WP, Anderson AJ, Kim YC, Pierson LS, Pierson EA, Lindow SE, Kobayashi DY, Raaijmakers JM, Weller DM, Thomashow LS, Allen AE, Paulsen IT 2012 Comparative genomics of plant-associated *Pseudomonas* spp.: insights into diversity and inheritance of traits involved in multitrophic interactions. PLoS Genet 8:e1002784. doi:10.1371/journal.pgen.1002784. 22792073 PMC3390384

[69] Kunst F, Ogasawara N, Moszer I, Albertini AM, Alloni G, Azevedo V, Bertero MG, Bessières P, Bolotin A, Borchert S, Borriss R, Boursier L, Brans A, Braun M, Brignell SC, Bron S, Brouillet S, Bruschi CV, Caldwell B, Capuano V, Carter NM, Choi SK, Cordani JJ, Connerton IF, Cummings NJ, Daniel RA, Denziot F, Devine KM, Düsterhöft A, Ehrlich SD, Emmerson PT, Entian KD, Errington J, Fabret C, Ferrari E, Foulger D, Fritz C, Fujita M, Fujita Y, Fuma S, Galizzi A, Galleron N, Ghim SY, Glaser P, Goffeau A, Golightly EJ, Grandi G, Guiseppi G, Guy BJ, Haga K, et al 1997 The complete genome sequence of the Gram-positive bacterium *Bacillus subtilis*. Nature 390:249–256. doi:10.1038/36786. 9384377

[70] Hayashi T, Makino K, Ohnishi M, Kurokawa K, Ishii K, Yokoyama K, Han CG, Ohtsubo E, Nakayama K, Murata T, Tanaka M, Tobe T, Iida T, Takami H, Honda T, Sasakawa C, Ogasawara N, Yasunaga T, Kuhara S, Shiba T, Hattori M, Shinagawa H 2001 Complete genome sequence of enterohemorrhagic *Escherichia coli* O157:H7 and genomic comparison with a laboratory strain K-12. DNA Res 8:11–22. 11258796 10.1093/dnares/8.1.11

[71] Bouchard D, Peton V, Almeida S, Le Marechal C, Miyoshi A, Azevedo V, Berkova N, Rault L, Francois P, Schrenzel J, Even S, Hernandez D, Le Loir Y 2012 Genome sequence of *Staphylococcus aureus* Newbould 305, a strain associated with mild bovine mastitis. J Bacteriol 194:6292–6293. doi:10.1128/JB.01188-12. 23105046 PMC3486374

[72] Tettelin H, Masignani V, Cieslewicz MJ, Eisen JA, Peterson S, Wessels MR, Paulsen IT, Nelson KE, Margarit I, Read TD, Madoff LC, Wolf AM, Beanan MJ, Brinkac LM, Daugherty SC, DeBoy RT, Durkin AS, Kolonay JF, Madupu R, Lewis MR, Radune D, Fedorova NB, Scanlan D, Khouri H, Mulligan S, Carty HA, Cline RT, Van Aken SE, Gill J, Scarselli M, Mora M, Iacobini ET, Brettoni C, Galli G, Mariani M, Vegni F, Maione D, Rinaudo D, Rappuoli R, Telford JL, Kasper DL, Grandi G, Fraser CM 2002 Complete genome sequence and comparative genomic analysis of an emerging human pathogen, serotype V *Streptococcus agalactiae*. Proc Natl Acad Sci U S A 99:12391–12396. doi:10.1073/pnas.182380799. 12200547 PMC129455

