# Supplementary material for: Impact of DNA Sequencing and Analysis Methods on 16S rRNA Gene Bacterial Community Analysis of Dairy Products
Source: mSphere. 2018 Oct 17;3(5):e00410-18. doi: 10.1128/mSphere.00410-18 (PMC6193606; doi:10.1128/mSphere.00410-18)
Supplement: TABLE S1 [file sph005182662st1.pdf]

Table S1

	QIIME 1	DADA2
**PCR amplicon mock community**		
Illumina paired-end	64,823 (0.05)[a]	39,056 (1.33)
Illumina single-end	41,263 (0)	81,011 (0)
Ion Torrent	170,611 (0.23)	196,270 (0.24)
**gDNA mock community**		
Illumina paired-end	53,615 (1.98)	35,130 (1.15)
Illumina single-end	38,337 (0.77)	75,601 (0.28)
Ion Torrent	203,486 (1.74)	181,323 (4.54)

[a] Each value represents the number of quality filtered reads and the percentage (in parenthesis) of chimeras from triplicate PCR amplicon and gDNA mock community samples.
