# Supplementary material for: Impact of DNA Sequencing and Analysis Methods on 16S rRNA Gene Bacterial Community Analysis of Dairy Products
Source: mSphere. 2018 Oct 17;3(5):e00410-18. doi: 10.1128/mSphere.00410-18 (PMC6193606; doi:10.1128/mSphere.00410-18)
Supplement: TABLE S2 [file sph005182662st2.pdf]

Table S2[a]

Taxonomy	Illumina paired-end		Illumina single-end	
	Percentage	BLASTn result[b]	Percentage	BLASTn result
Adlercreutzia	NA[c]	NA	1 (0.02)	Exact
Allobaculum	2 (0.04)[d]	Exact	1 (0.04)	Exact
Anaeroplasma	1 (0.09)	1 off	1 (0.04)	1 off
Bacteria	1 (0.00)	NA	8 (0.55)	Exact
Bacteroidales	NA	NA	1 (0.02)	3 off
Bacteroides	1 (0.09)	Exact	1 (0.04)	Exact
Candidatus Arthromitus	1 (0.07)	Exact	1 (0.04)	Exact
Clostridia	NA	NA	1 (0.04)	3 off
Clostridiales	27 (3.85)	Exact	15 (1.73)	Exact
Coprobacillus	1 (0.04)	Exact	1 (0.02)	Exact
Coprococcus	3 (0.20)	1 off	1 (0.02)	Exact
Coriobacteriaceae	2 (0.15)	Exact	2 (0.04)	Exact
Dehalobacterium	1 (0.07)	Exact	1 (0.02)	Exact
Desulfovibrio	1 (0.16)	Exact	1 (0.09)	Exact
Desulfovibrionaceae	2 (0.13)	Exact	3 (0.18)	Exact
Dorea	1 (0.09)	Exact	1 (0.04)	Exact
Erysipelotrichaceae	1 (0.02)	Exact	1 (0.04)	Exact
Firmicutes	NA	NA	2 (0.18)	Exact
Helicobacter	2 (0.04)	Exact	1 (0.02)	Exact
Helicobacteraceae	1 (0.13)	Exact	1 (0.11)	Exact
Lachnospiraceae	17 (1.81)	Exact	24 (1.68)	Exact
Lactobacillaceae	1 (0.97)	Exact	1 (0.44)	Exact
Lactobacillus	4 (2.88)	Exact	4 (1.64)	Exact
Mogibacteriaceae	1 (0.02)	Exact	NA	NA
Mucispirillum	NA	NA	1 (0.02)	Exact
Mycoplasmataceae	2 (0.97)	6 off	1 (0.51)	3 off
Oscillospira	10 (2.97)	Exact	9 (1.86)	Exact
Prevotella	1 (0.02)	5 off	NA	NA
Prevotellaceae	NA	NA	1 (0.02)	Exact
RF39	2 (0.02)	Exact	1 (0.02)	Exact
Rickettsiales	1 (0.02)	2 off	NA	NA
Rikenella	1 (0.02)	4 off	NA	NA
Rikenellaceae	3 (0.20)	Exact	3 (0.11)	Exact
Roseburia	1 (0.02)	Exact	NA	NA
Ruminococcaceae	9 (0.89)	Exact	9 (0.69)	Exact
Ruminococcus	8 (0.62)	Exact	6 (0.22)	Exact

S24-7	4 (0.44)	Exact	4 (0.51)	Exact
Sutterella	1 (0.07)	Exact	1 (0.05)	Exact
Turicibacter	2 (2.44)	Exact	1 (1.26)	Exact
**Sum**	116 (19.53)		111 (12.27)	

[a] Results were based on DADA2 analysis pipeline with Greengenes database rarefied at 5483 reads per sample.

[b] Exact represents that there is 100% match between the ASV sequence and the sequences in the NCBI nr/nt database. Each number followed by "off" represents that there are the number of mismatched bases between the ASV sequences and the best matched sequences in the NCBI nr/nt database.

[c] NA: Not available. The ASV does not exist for the particular Illumina MiSeq read analysis.

[d] Each value represents the number of ASVs and mean percentage (in parenthesis) of that taxon from triplicate gDNA mock community samples. The sum reflects the % of all spurious reads and not just the most abundant ASVs represented in Table 5.
